# Intersexual differences of heat shock response between two amphipods (*Eulimnogammarus verrucosus* and *Eulimnogammarus cyaneus*) in Lake Baikal

**DOI:** 10.7717/peerj.2864

**Published:** 2017-02-21

**Authors:** Daria Bedulina, Michael F. Meyer, Anton Gurkov, Ekaterina Kondratjeva, Boris Baduev, Roman Gusdorf, Maxim A. Timofeyev

**Affiliations:** 1 Institute of Biology, Irkutsk State University, Irkutsk, Russia; 2 School of the Environment, Washington State University, Pullman, WA, USA; 3 Baikal Research Centre, Irkutsk, Russia; 4 University of Notre Dame, Notre Dame, United States

**Keywords:** Hsp70, Proteome, Amphipoda, Freshwater benthic invertebrates, Stress response, Two-dimensional gel electrophoresis, Thermotolerance, Baikal, *Eulimnogammarus verrucosus*, *Eulimnogammarus cyaneus*

## Abstract

Acute temperature fluctuations are common in surface waters, and aquatic organisms may manifest physiological responses to punctuated temperature spikes long before behavioral responses. Ectotherms, especially cryophilic stenotherms such as those endemic to Lake Baikal (Siberia), may demonstrate specialized physiological responses to acute temperature increases because their proteomes have evolved to function most efficiently at lower temperatures (e.g., <10 °C). Therefore, our study questioned the nature and degree of variation in physiological response to acute thermal stress in two congenerous, endemic Baikal amphipod species, *Eulimnogammarus verrucosus* and *Eulimnogammarus cyaneus*. We hypothesized that because interspecific and intersexual thermosensitivity varies significantly among ectotherms, there would be divergent intersexual and interspecific strategies to withstand acute thermal stress, manifested in different protein compositions and concentrations. We exposed individuals to the species’ respective LT50 for one hour followed by a three-hour recovery period. We then performed 1D-PAGE, Western blotting, 2D-PAGE, and Mass Spectrometry techniques and assessed relative intersexual and interspecific changes in proteomic composition and heat shock protein 70 level. Our results demonstrate that females tend to be more sensitive to an acute thermal stimulus than males, most likely because females allocate significant energy to reproduction and less to heat shock response, evidenced by females’ significantly lower LT50_time_. Lower level of Hsp70 was found in females of the thermosensitive *E. verrucosus* compared to males of this species. No intersexual differences were found in Hsp70 level in thermotolerant *E. cyaneus*. Higher levels of hemocyanin subunits and arginine kinase were found in *E. cyaneus* females after heat shock and recovery compared to males, which was not found for *E. verrucosus*, suggesting interspecific mechanisms for *E. cyaneus*’s higher thermotolerance. These differing responses between species and sexes of Baikal amphipods may reflect more general strategies for maintaining homeostatic conditions during acute thermal stress. As mean surface water temperatures increase worldwide, the net efficiency and efficacy of these strategies could give rise to long term changes in physiology, behavior, and interactions with other species, potentially precipitating population and community level alterations.

## Introduction

Temperature, as an abiotic factor, has the potential to alter physiology (Hochachka & Somero, 2002; Somero, 2012; Tomanek, 2010), behavior (Huey, 1991), and species interactions (e.g., Dell, Pawar & Savage, 2014). While increased ambient air temperatures create warmer average temperatures, surface waters such as lakes have likewise warmed worldwide (O’Reilly et al., 2015), thereby creating a warmer habitat for the organisms that live within them. The ability of an immediate adaptation or stress-response is essential for aquatic ectotherms that are unable to self-regulate their core body temperature. Cellular responses to acute thermal change (heat shock) are evolutionarily conserved mechanisms that are designed to preserve protein structure during times of thermal stress (Kültz, 2003). Among the many proteins involved in these processes, heat shock proteins (Hsps) are integral to protecting cellular proteins during proteotoxic stress and promote protein refolding (Feder & Hofmann, 1999; Vabulas et al., 2010). Hsp70 is a well-studied member of the Hsp family (Mayer & Bukau, 2005), which is responsible for the adaptation of poikilothermic organisms to adverse environmental conditions (Feder & Hofmann, 1999; Evgen’ev et al., 2007). Despite Hsp70 acting as a universal protective mechanism against various stressors, its synthesis is metabolically expensive and is not involved in the stress-response until absolutely necessary (Hofmann & Somero, 1995). In addition to Hsp70 and other molecular chaperones, Kültz (2003) defined several major proteomic components of cellular stress response, including redox regulation, DNA repair/chromatin stability, cell cycle, protein degradation and translation/protein synthesis. Although cellular stress response pathways are highly constrained, they can vary in phylogenetically distant organisms (Trapp et al., 2014). This is important to consider for uniquely isolated ecosystems with a high rate of endemic speciation and biodiversity, such as Lake Baikal in Siberia. Lake Baikal boasts exceptional biodiversity with a wide range of endemic flora and fauna, a large portion of which are well adapted to cold-water conditions with an average surface temperature of 1 °C in the winter and 8 °C in the summer (Hampton et al., 2008). Amphipods constitute the most diverse taxonomic group in Lake Baikal (Amphipoda, Crustacea), comprising 354 described species and sub-species (Takhteev, Berezina & Sidorov, 2015).

The vast majority of the endemic amphipods of Lake Baikal evolved under stable environmental conditions, including cold temperatures, low nutrient levels, high dissolved oxygen, and low mineral conditions (Mats, Shcherbakov & Efimova, 2011). The current abiotic conditions both around and in Lake Baikal continue to change rapidly, with Lake Baikal’s surface waters warming approximately twice as fast as the ambient air temperatures, which are increasing at a rate of 0.2 °C decade^−1^ (Hampton et al., 2008; Shimaraev et al., 2002). Although no studies have attempted to project spatial niche alterations Baikal amphipods, Jakob et al. (2016) demonstrated that *Eulimnogammarus verrucosus* and *Eulimnogammarus cyaneus* enter a state of pejus, the progressively deleterious temperature range, coupled with the onset of PO_2_ in the arterial blood due to reduced ventilator or cardiac performance (Frederich & Pörtner, 2000), at temperatures of 10.6 °C and 19.1 °C, respectively. With temperatures increasing rapidly in Lake Baikal, our work, therefore, focuses on the potential for protein-level responses following exposure to increasing temperatures.

Many studies have already demonstrated that the response to abiotic stressors, such as temperature, vary with biological factors, such as sex (Madeira et al., 2012; Sornom et al., 2010; Vaughn, Turnross & Carrington, 2014). These deviations are often the result of differences in the physiology and energy allocations between males and females (Janowitz & Fischer, 2011; Nguyen, Bressac & Chevrier, 2012). The differing sensitivities between the sexes to thermal shifts can propagate through biological levels, eventually altering population and community composition and abundance. To provide comprehensive information about the potential physiological responses of species to the effects of a changing climate, studies of intraspecific differences in thermotolerance are essential. However, there is currently a lack of such research for stenophilic cold-water species, such as those found in Lake Baikal, that may show the greatest response to a warming environment (Moore et al., 2009).

In this pursuit, our work attempts to understand the molecular responses of two coldwater amphipods to acute thermal stress. The main objectives of our study were therefore two-fold: (1) to analyze intersexual variations of thermal sensitivity and Hsp70 levels and (2) to compare proteomic heat shock responses in the two congener Baikal littoral amphipods, *E. cyaneus* and *E. verrucosus*. These species were selected for study because they are the most abundant species in the littoral zone of Lake Baikal (Timoshkin et al., 2009) and are considered potential bioindicators for monitoring the benthic community health (Timofeyev et al., 2010). We hypothesized that because of the intersexual variation in the amphipods’ physiology, which is common in invertebrate species (e.g.,  Gilbert & Williamson, 1983; Gross et al., 2001), females and males will have differences in thermal sensitivity, which will be reflected in their respective responses at the cellular level.

## Material and Methods

### Experimental animals

*Eulimnogammarus verrucosus* (Gerstfeldt, 1858) is a common, endemic species in Lake Baikal, largely inhabiting the littoral and sub-littoral zones (e.g., 0–15 m) (Kravtsova et al., 2003). *E. verrucosus* is a stenobiotic species, reproducing during the winter when temperatures are lowest (0−6 °C). Females with eggs appear at the end of October, and juveniles appear at the end of May (Gavrilov, 1949). At that time, the juveniles are found at the upper littoral zone, but adults migrate to the deeper parts of the littoral zone (Weinberg & Kamaltynov, 1998). This species is thermosensitive and stenothermal (Bedulina et al., 2013), and in laboratory experiments, the adults preferred a narrow temperature zone of 5–6 °C (Timofeyev & Shatilina, 2007).

*Eulimnogammarus cyaneus* (Dybowsky, 1874) is a small (11–15 mm), endemic amphipod and is representative of the upper littoral zone. This species inhabits the rocky shore of the lake with 90% of its population density at the narrow zone of the water edge (Bazikalova, 1941). Reproduction takes place three times during the summer, starting May, when females first appear with eggs (Gavrilov, 1949). This species has an enhanced thermal response compared to *E. verrucosus* (Bedulina et al., 2013) and prefers temperatures approximately 11–12 °C (Timofeev & Kirichenko, 2004).

### Sampling and experimental procedures

Amphipods were collected in three consecutive years (2013–2015) with a hand net in the littoral zone (0–0.5 m) during each species’ respective mating time (*E. cyaneus* in June and *E. verrucosus* in November) near the coastal town of Listvyanka (51°84′85″N, 104°88′37″E). The animals were transferred to the laboratory in thermostatic boxes. Only specimens in amplexus were selected for further study because, in this state, it was possible to determine the sex of amphipods without too much manipulation under the microscope (i.e., the upper specimen in the amplexus was always male). The females from both species were on the second or third stages of their molting cycle (Bazikalova, 1941; with developed but hairless oostegites or with eggs in the marsupium). The males and females were carefully divided from the amplexus and were separately allowed to acclimate in 2-L glass aquariums with constantly aerated lake water for five days at 6–7 °C (average annual temperature of Baikal littoral; Weiss, Carmarck & Koropalov, 1991; Falkner et al., 1991) to eliminate the potential seasonal variations in physiology. During the acclimation period, the amphipods were fed with the commercial food TetraMin (Tetra, Germany) and a dried mix of algae and amphipods from their environment. The water was changed every other day. No mortality was observed during the acclimation.

To determine the relative sensitivity to a heat shock stimulus, five replicates of each sex and amphipod species were incubated in 1-L glass aquaria. Individual sexes and species were then placed separately in constantly aerated lake water at 25 °C for *E. verrucosus* and 26 °C for *E. cyaneus* due to the difference in the thermotolerance of these two species (Bedulina et al., 2013). Mortality was monitored each hour.

To determine the heat shock response, five replicates of each sex and species were incubated in 1-L glass aquaria with well-aerated lake water, which was preheated to the previously determined temperature to cause 50% mortality of the mixed adult population (24.5 °C for *E. verrucosus* and 25.5 °C for *E. cyaneus* Bedulina et al., 2013). The individuals were exposed to heat shock for one hour, after which, they were transferred to a separate tank at 6−7 °C for a 3 h recovery period. Following the recovery, the animals were fixed by flash freezing with liquid nitrogen. The control group was fixed under the pre-acclimation (6−7 °C) conditions before the experiment.

### 1D-PAGE and Western blotting

The protein isolation for the sodium dodecyl sulphate-polyacrylamide gel electrophoresis (SDS-PAGE) was performed as described previously in Bedulina et al. (2013). Two specimens of *E. verrucosus* and four specimens of *E. cyaneus* (to compensate for the differences in size) were used for protein isolation. SDS-PAGE was performed in a 70 × 80 × 0.75 mm^3^ gel blocks (Laemmli, 1970) using a Mini-PROTEAN II Electrophoretic Cell (BIO-RAD, Hercules, CA, USA) apparatus for electrophoresis in a 10% polyacrylamide gel. Forty-five micrograms of total protein were loaded to determine both Hsp70 and actin expression. The blotting to a polyvinylidene difluoride transfer membrane (GE Healthcare, Amersham, UK) was performed via a semi-dry transfer according to Towbin, Staehelin & Gordon (1979) in the Trans-Blot^®^ SD Semi-Dry Transfer Cell (BIO-RAD, Hercules, CA, USA) apparatus. Equal loading of the protein was verified by staining the membranes with 0.5% Ponceau Red in 1% acetic acid. The bovine monoclonal anti-Hsp70 antibody (produced in mouse; Sigma-Aldrich, # H5147, dilution 1:1,000) and an alkaline phosphatase-conjugated secondary antibody (anti-mouse IgG:AP Conj., Stressgen # SAB-101, dilution 1:1,000) were used for Hsp70 detection. The chicken anti-actin antibody (produced in rabbit; # A2668, dilution 1:1,000; Sigma-Aldrich) and a secondary antibody (anti-rabbit IgG, # A9919, dilution 1:1,000; Sigma) were used for the detection of *β*-actin. The protein-antibody complexes were detected using 5-Bromo-4-chloro-3-indolyl phosphate disodium salt and Nitrotetrazolium Blue chloride. Hsp70 and actin levels were measured by semi-quantitative analysis of gray values on scanned Western blot membranes using “ImageJ” software with the “Fiji” package (Schindelin et al., 2012). The levels of Hsp70 were normalized relative to *β*-actin expression in each sample.

### 2D-PAGE and Mass Spectrometry

Three *E. verrucosus* and ten *E. cyaneus* were ground under liquid nitrogen into a thin powder with the addition of 0.1 M Tris–HCl, pH 7.6 and 1% of phenylmethylsulfonyl fluoride (PMSF) and 1% of protease inhibitor cocktail (Amresco). The homogenates were centrifuged at 4 °C and 7,500 g for 15 min to remove the insoluble part of the homogenate. The protein concentration was measured by the method as described by Bradford (1976). One milligram of total protein in each sample was taken for further purification. The proteins were precipitated with the addition of 10% trichloric acid and pellets were washed twice with 96% cold ethanol, resuspended in 0.1 M Tris–HCl, pH 7.6 with 1% of PMSF and 1% of protease inhibitor cocktail (Amresco) and re-precipitated with two volumes of cold acetone. The dried pellets were diluted in a lysis buffer containing 8 M urea, 2% Triton X-100, 0.05 M dithiothreitol, 1% of a protease inhibitor cocktail (Amresco) and ampholytes 3.5–10 (BioRad) (O’Farrell, 1975). The two-dimensional gel electrophoresis was performed as described previously (O’Farrell, 1975) with modifications. Isoelectric focusing (IEF) was carried out in a modified system BioRad PROTEAN II xi cell (BioRad). Custom made glass capillary tubes with an inner diameter of 0.4 cm and a gel length of 13.5 cm (3.84% polyacrylamide gel with addition of ampholytes 3.5–10 and 5–7 (BioRad)) were loaded with 500 µg of the purified protein. IEF was conducted for 18 h with the following gradual rising of voltage: 100 V (1 h)–200 V (1 h)–300 V (1 h)–400 V (1 h)–500 V (1 h)–600 V (1 h)–700 V (10 h)–900 V (2 h). Subsequently, the IEF gels were extracted and incubated for 1 h with Laemmli buffer (Laemmli, 1970; 65 mM Tris–HCl, pH 6.8, 10% glycerol, 2% sodium dodecyl sufate, 4% *β*-mercaptoehanol) and frozen at −80 °C. To separate the proteins by molecular weight, SDS-PAGE was performed in 160 × 200 × 1 mm 10% polyacrylamide gel blocks in a BioRad PROTEAN II xi cell (BioRad, USA). The gels were stained in a 0.2% solution of Coomassie Brilliant Blue G250 (CBB) in 25% isopropanol and 10% acetic acid for 20 min followed by double destaining in 25% isopropanol and 10% acetic acid for 30 min. To finalize the destaining, the gels were placed into distilled water overnight. Pictures of the gels were obtained using MiniLumi system (Berthold Technologies). The gel image analysis was carried out with “ImageJ” software with the “Fiji” package (Schneider, Rasband & Eliceiri, 2012), using the custom-made add-on for 2D gel images (Gurkov, Kondratyeva & Bedulina, 2014). The gray values of each selected spot on the gels were normalized relative to the cumulative level of two spots, related to *β*-actin (spots numbers: 19, 20) for each gel.

The protein spots were excised and digested in the gel with modified trypsin (Promega). Mass spectrometry was then performed with a matrix-assisted laser desorption/ionization time-of-flight/time-of-flight tandem (MALDI-TOF/TOF) mass-spectrometer UltrafleXtreme BrukerDaltonics (BRUKER, Germany). The peak list generation and protein identification were made using the FlexAnalysis 3.3 (Bruker Daltonics, Bremen, Germany) software and sequence databases of the National Center for Biotechnology Information (NCBI-nr) and SwissProt, using both the complete database and one restricted to “Invertebrates” and “Other Metazoa” with the Mascot search engine (http://www.matrixscience.com). The search parameters were as follows: peptide tolerance 30 ppm; fixed modification: carbamidomethylation of cysteines; and variable modification: oxidation of methionine. The mass spectra were recorded at the Human Proteome Shared Facility Centre in the Institute of Biomedical Chemistry, Moscow, Russia.

### Data analyses

All of the exposure experiments were repeated 3–5 times. Normality was tested by the Kolmogorov–Smirnov test, and equal variance was assessed with Levene’s test. All of the data were found to satisfy the assumptions of normality and homoscedasticity. The data from the Western blot were analyzed via a one-way analysis of variance (ANOVA). A post-hoc analysis was conducted with a Bonferroni-corrected *t*-test. The data from the 2D-gel images were analyzed using a paired *t*-test for each sex of each species. Differences were considered to be significant at values of *P* < 0.05. All of the statistical analyses were carried out using SigmaStat software (version 3.11, Aspire Software International, Ashburn, VA, USA). The data are expressed as the means ± standard deviations.

The mortality data were fitted to the Weibull model (Wilson, 1994) in R (R Core Team, 2016), and the LT50 (the temperature at which the mortality of 50% of individuals occurred) values derived from it:

\begin{eqnarray*}\begin{array}{@{}llll@{}} \displaystyle m=100- \frac{100}{{e}^{( \frac{t}{p} )^{r}}} &\displaystyle &\displaystyle &\displaystyle \text{} \end{array} \end{eqnarray*}

where *m* –cumulative portion of dead individuals, %; *t* –temperature, °C; *p* and *r* − regression coefficients.

## Results

### Thermotolerance

**Figure 1:**
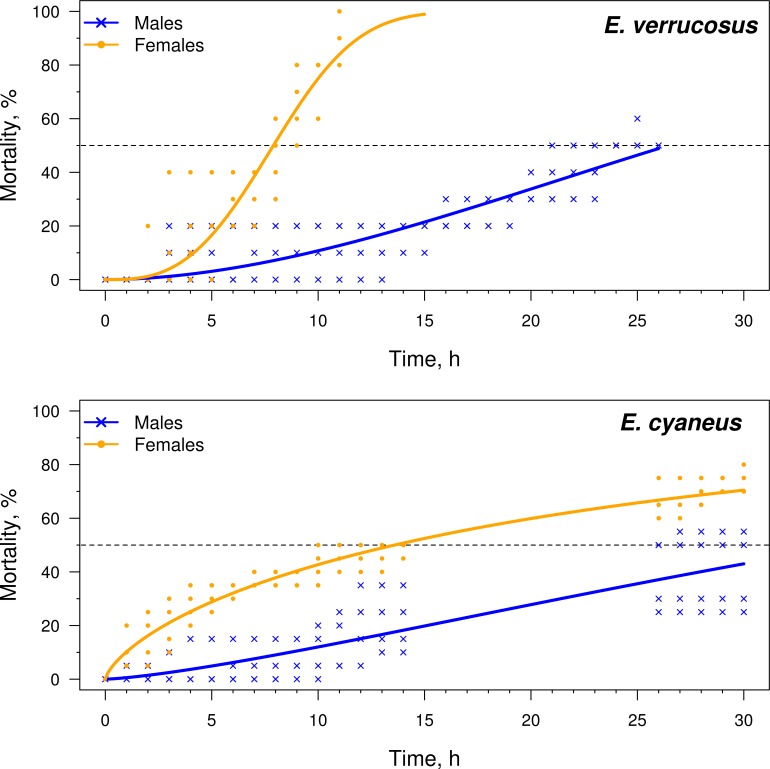
Cumulative mortality curves of the male and female *E. verrucosus* and *E. cyaneus* exposed to 24.5 °C (for *E. verrucosus*) and 26.5 °C (for *E. cyaneus*), *n* = 5.

The females from both species demonstrated a higher sensitivity to heat shock than the males. For *E. cyaneus*, the LT50_time_ was calculated as 13.6 h for females and 35 h for males. For *E. verrucosus*, LT50 was estimated as 7.9 h for females, while the males lived significantly longer (e.g., LT50 = 26.5 h) (Fig. 1).

**Figure 2:**
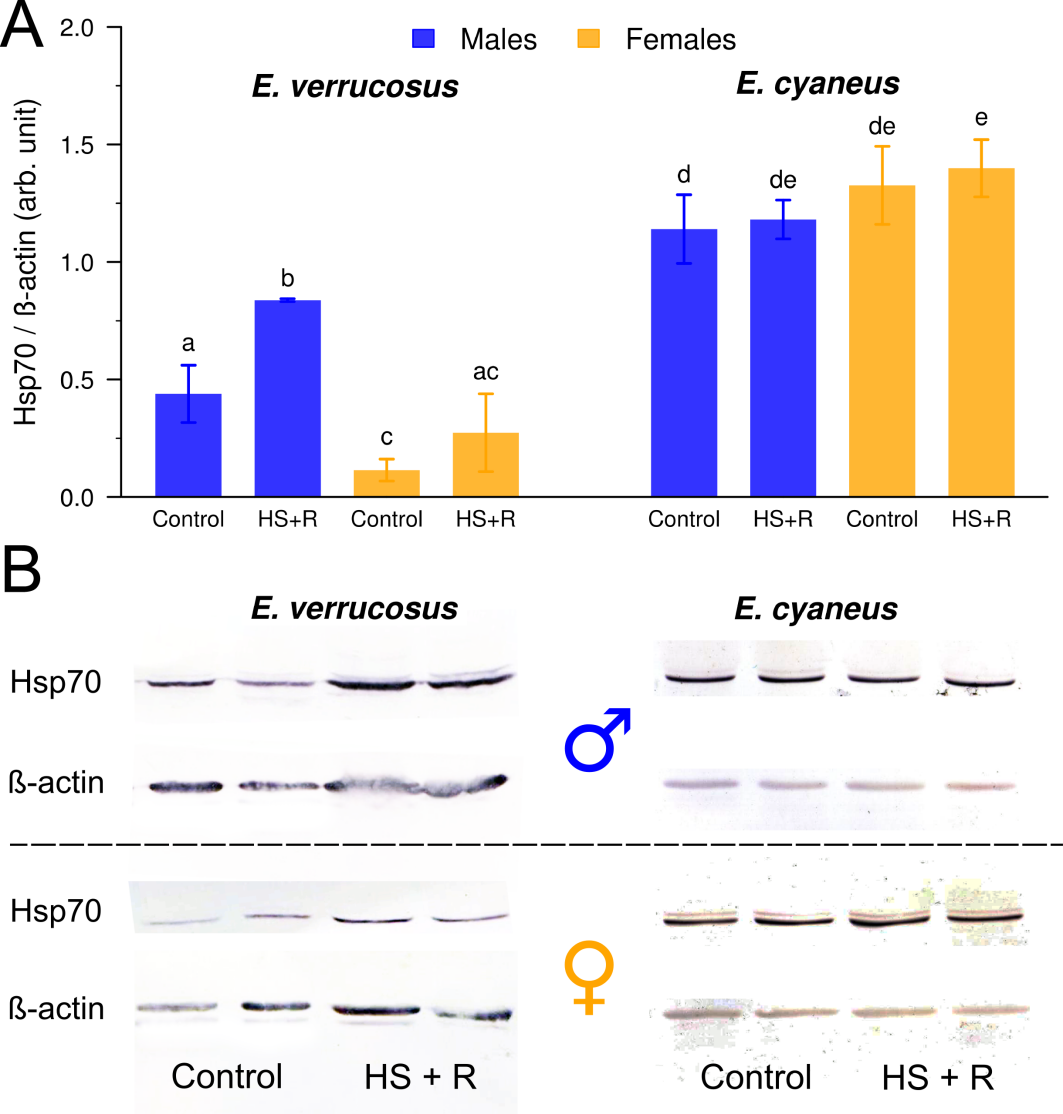
Relative levels of Hsp70 in male and female *E. verrucosu*s and *E. cyaneus* exposed to the heat shock temperatures (species-specific LT50) for 1 h and the subsequent recovery (at 6°C) for 3 h (HS + R), *n* = 5. Representative membranes are shown (B), and the densitometry analysis (A), where the levels of Hsp70 are normalized to the *β*-actin levels in each sample.

### Heat shock proteins

The basal levels of Hsp70 in the controls for each of the two species were significantly different. The Hsp70 level was 2.6 times higher in the male *E. cyaneus* than in the male *E. verrucosus* (*P* < 0.001). Females *E. cyaneus* contained 11.6 times higher Hsp70 levels than female *E. verrucosus* (*P* < 0.001). The heat shock (1 h) and recovery (3 h) significantly induced Hsp70 accumulation in the *E. verrucosus* males (*P* = 0.01). A slight tendency of Hsp70 induction was also observed in the *E. verrucosus* females; however, this increase was not statistically significant (Fig. 2). The opposite was observed for the thermotolerant *E. cyaneus*. A slightly higher basal level of Hsp70 was found in the female controls compared to the males. This higher level was elevated as the result of an additional isoform of Hsp70, which is expressed to a higher extent in females (Fig. 2). No significant increase in Hsp70 was found in this species when it was exposed to heat shock (1 h) and the subsequent recovery (3 h). However, the stress-induced level of Hsp70 in the females was significantly higher than the control level in the males (*P* = 0.026) (Fig. 2).

### Sexual dimorphism of 2D-proteomic profiles

To further characterize the intersexual properties of the proteomic stress response, the 2D-PAGE protein patterns were analyzed for protein presence and relative abundance. The analysis of the CBB stained gels detected a maximum of 317 protein spots from *E. cyaneus* and a maximum of 201 protein spots from *E. verrucosus*.

**Figure 3:**
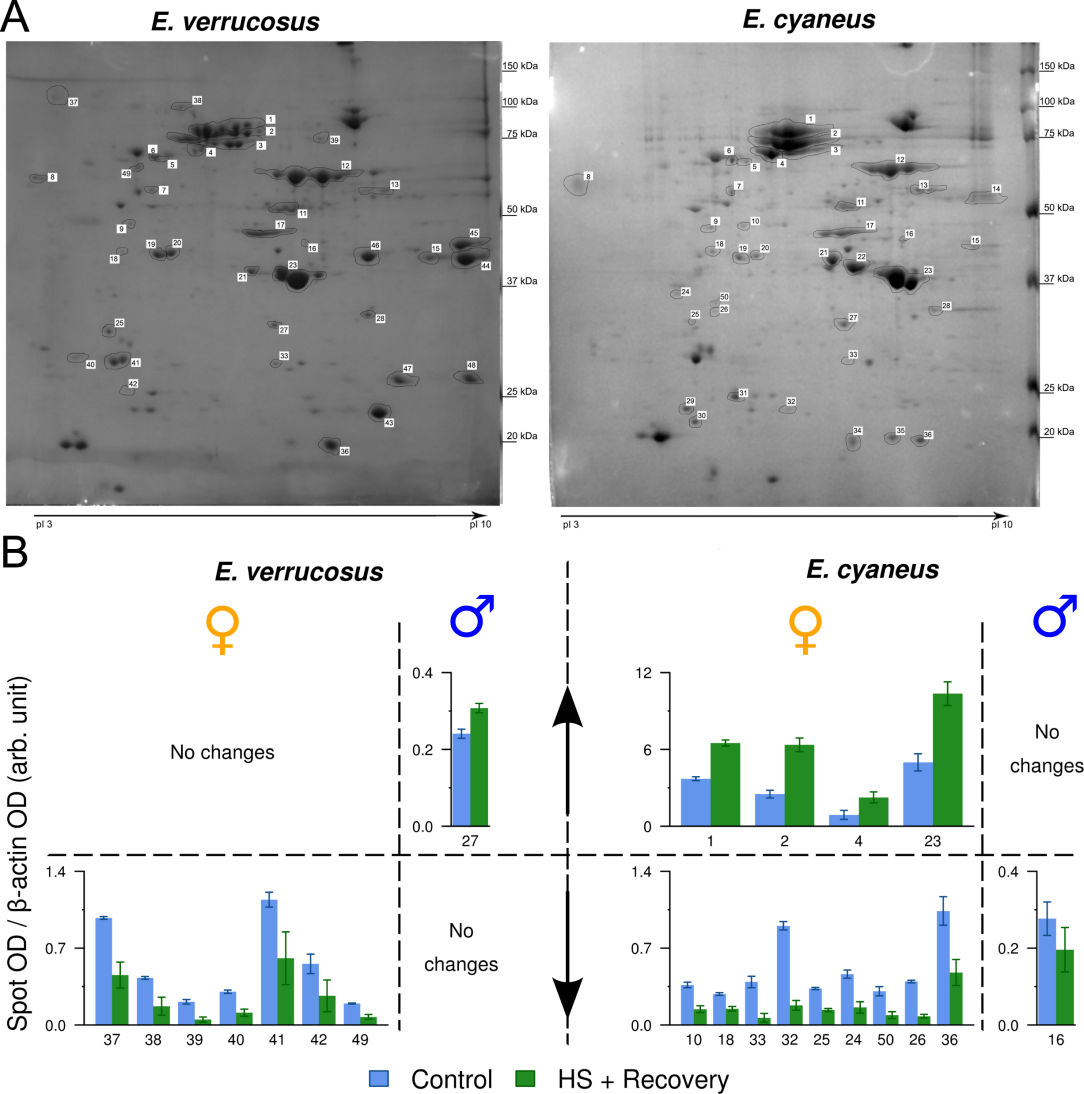
2D-PAGE of the total protein from the male and female *E. verrucosu*s and *E. cyaneus* exposed to heat shock (LT50, 1 h) and the subsequent recovery (6 C, 3 h) (*n* = 3–5). Representative gels are shown (A). The gray values of each protein spots were normalized relative to the gray values of *β*-actin (B, spots n 19, 20).

Up to 15 differently expressed protein spots were found in the gels from males and females of both studied species. No significantly different male-specific protein spots were found on gels. The female proteomes of both species were characterized by the presence of large protein spots with a molecular weight (MW) of 66 kDa and an isoelectric point (pI) of 5.6–5.8 (Fig. 3. spot number 12). Figure 3 and Table 1 further detail the various spots observed for female *E. cyaneus* and *E. verrucossus*.

**Table 1:** Female specific protein spots on the 2D-PAGE gels in *E. verrucosus* and *E. cyaneus*. The location, identification and pI/MW values of the spots are indicated on the Fig. 3 and the Table 2.

Spot number (Fig. 3)	*E. cyaneus* ♀	*E. verrucosus* ♀
12	+	+
15	+	+
22	+	
29	+	
30	+	
31	+	
34	+	
35	+	
36	+	+
43		+
44		+
45		+
46		+
47		+
48		+

Heat shock and the subsequent recovery altered the optical density (OD) of 13 protein spots in *E. cyaneus* females. Among these, nine protein spots had a significantly decreased OD, and four were significantly increased after the exposure. Only the OD for one protein spot decreased on the gels containing the *E. cyaneus* males. Seven protein spots had a significantly decreased OD among the female *E. verrucosus,* whereas, in males only one protein spot showed a slightly increased OD (Fig. 3).

### Protein identification

Using public databases (NCBInr, SwissProt), we determined the identity of 18 protein spots in the gels. In all of the experimental conditions, the most abundant proteins on the CBB-stained gels were represented by hemocyanin, enolase, the tubulin beta chain, actin and arginine kinase. Among the identified proteins, hemocyanin and arginine kinase had an increased OD in the *E. cyaneus* females, and cytosolic malate dehydrogenase was increased in the *E. verrucosus* males that were exposed to heat shock and recovery. Due to the limited number of Amphipoda sequences in publicly accessible databases, it was not possible to identify all of the protein spots (Table 2).

**Table 2:** Identification of the selected spots on the 2D-PAGE gels in *E. verrucosus* and *E. cyaneus*.

Number	pI (on gels)	MW, kDa (on gels)	Identification	Score
**1**	**5.05–5.29**	**85.0**	**gi—62484806, hemocyanin subunit 1 [*Gammarus roeseli*]**	**95**
**2**	**5.05–5.29**	**77.0**	**gi—62484806, hemocyanin subunit 1 [*Gammarus roeseli*]**	**80**
**3**	**5.05–5.29**	**71.5**	**gi—62484806, hemocyanin subunit 1 [*Gammarus roeseli*]**	**65**
**4**	**5.2**	**71.2**	**gi—62484806, hemocyanin subunit 1 [*Gammarus roeseli*]**	**39**
**5**	**5.0**	**67.5**	**gi—74274968, heat shock protein 70 [*Pachygrapsus marmoratus*]**	**111**
**6**	**5.0**	**67.5**	**gi—324604904, heat shock protein 70 [*Marsupenaeus japonicus*]**	**135**
**7**	**5.0**	**66.2**	**gi—307199045, 60 kDa heat shock protein, mitochondrial [*Harpegnathos saltator*]**	**142**
**8**	**4.3**	**62.8**	**EH269494, EH269494.1, Cellobiogydralase, *Gammarus pulex***	**70**
9	5.0	48.0	Unidentified	
10	5.1	48.0	Unidentified	
**11**	**5.5**	**54.0**	**ACO12036.1, tubulin beta chain [*Lepeophtheirus salmonis*]**	**50**
12	5.7–5.9	66.0	Unidentified	
**13**	**5.8**	**60.0**	**gi—298398818, catalase [*Heliconius melpomene melpomene*]**	**70**
14	9.0	58.0	Unidentified	
15	6.1	47.0	Unidentified	
16	5.7	46.0	Unidentified	
**17**	**5.5**	**47.0**	**UniProtKB/Swiss-Prot: P56252.1, enolase, *Homarus gammarus***	**48**
18	5.0	46.0	Unidentified	
**19**	**5.0**	**42.1**	**gi—3907620, actin 1 [*Penaeus monodon*]**	**198**
**20**	**5.1**	**42.5**	**gi—3907620, actin 1 [*Penaeus monodon*]**	**150**
21	5.4	42.0	Unidentified	
**22**	**5.5**	**40.6**	**EH275721, EH275721.1, *Gammarus pulex*, fructose 1,6-bisphosphate aldolase**	**98**
**23**	**5.8**	**36.9**	**gi—328679650, arginine kinase [*Pselactus* sp. BHJ-2011]**	**135**
**24**	**4.7**	**37.5**	**gi—162286975, tropomyosin [*Oratosquilla oratoria*]**	**238**
25	4.8	33.5	Unidentified	
26	4.9	35.0	Unidentified	
**27**	**5.5**	**34.1**	**gi—307644679, cytosolic malate dehydrogenase [*Phyllocnistis labyrinthella*]**	**70**
**28**	**6.1**	**36.7**	**gi—190014502, putative glyceraldehyde-3-phosphate dehydrogenase [*Gammarus locusta*]**	**97**
29	4.8	27.2	Unidentified	
30	4.8	26.3	Unidentified	
31	5.0	28.1	Unidentified	
32	5.2	27.0	Unidentified	
33	5.5	30.8	Unidentified	
34	5.5	25.4	Unidentified	
35	5.7	25.6	Unidentified	
36	5.8	25.7	Unidentified	
37	5.0	130.0	Unidentified	
38	5.1	100.0	Unidentified	
39	5.6	78.3	Unidentified	
40	4.5	33.5	Unidentified	
41	4.8	30.8	Unidentified	
42	5.0	28.2	Unidentified	
43	5.8	25.0	Unidentified	
44	9.0	45.0	Unidentified	
45	9.0	46.0	Unidentified	
46	6.1	34.1	Unidentified	
47	6.1	28.2	Unidentified	
48	9.0	28.0	Unidentified	
49	5.0	65.5	Unidentified	
50	4.9	36.0	Unidentified	

## Discussion

This study presents an investigation of sexual dimorphism in acute thermal stress sensitivity and cellular stress response mechanisms in two closely related Baikal endemic amphipods, which differ in thermotolerance. As shown by Hoback & Barnhart (1996) and Sornom et al. (2010), female gammarids are more sensitive to environmental challenges, such as hypoxia and salinity, than males. Our results clearly demonstrate an enhanced sensitivity of females to acute thermal stress in the two species of endemic amphipods in Lake Baikal. Such a sensitivity can be explained by the increased metabolic energy demand for oogenesis compared to the less energy demanding spermatogenesis (Buikema Jr & Benfield, 1979). In this case, the energy resources used for the stress response might be limited in females. Sprague (1963) reported different thermal sensitivities in the male and female amphipods *Hyalella azteca* (Saussure, 1858), *Gammarus fasciatus* Say, 1818, and *Gammarus pseudolimnaeus* Bousfield, 1958, which depended on temperatures of acclimation; when they were acclimated at 20 °C, the female amphipods showed a greater sensitivity to acute thermal stress than the males.

Gammarids are known to vary in intraspecific sensitivity to environmental stressors. Females were shown to be more sensitive to chemical pollutants (McCahon & Pascoe, 1988; Schill, Görlitz & Köhler, 2003) salinity (Ashton, 2006) and hypoxia and anoxia (Hoback & Barnhart, 1996). Other works indicate the opposite where males demonstrate higher sensitivity to pollutants (Gismondi, Beisel & Cossu-Leguille, 2012). Additionally, no differences in survival of sexes were detected under some environmental stressors, such as thermal and chemical stress (Barros et al., 2017; Foucreau et al., 2014). These discordances may be explained by differing molting stages among females (ovigorous, non-ovigorous), molting frequency, and a size-effect. Another important point could be the different endpoints, which are used to define sensitivity. Barros et al. (2017) demonstrated no intersexual differences in survival of *Gammarus locusta* (Linnaeus, 1758), exposed to triclocarban; however, females expressed a behavioral reaction and a higher level of lipid peroxides, indicating cellular stress. Additionally, sensitivity to acute stressors, like heat shock, can contrast with respective chronic exposure.

One potential explanation for the different sensitivities of the males and females is the activation of stress-response mechanisms. The activity of molecular chaperones, such as Hsp70, is a universal cellular mechanism, protecting cells against a variety of proteotoxic stresses, including heat shock (Mayer & Bukau, 2005). Upregulation of Hsp70 accumulation and their genetic expression have been found in many aquatic ectotherms (Feder & Hofmann, 1999). It was shown that this response depends on the taxonomic status and evolutionary history of a species, such as many Antractic species lack proper regulation of inducible forms of Hsp70 expression (Clark & Peck, 2009; Peck, 2016). Species-specific differences in Hsp70-mediated stress responses and the underlying molecular mechanisms of gene expression regulation were previously described for both of the studied amphipod species (Bedulina et al., 2013). The thermotolerant species *E. cyaneus* has a significantly higher basal level of Hsp70 than the thermosensitive *E. verrucosus*, as well as a lower rate and a later onset of Hsp70 accumulation during heat shock. The elevated thermotolerance of *E. cyaneus* is associated with a higher number of *hsp70* copies in the genome, more compact promoters of *hsp70* genes and more stable complexes of heat shock factors and heat shock elements in the *hsp70* genes (Bedulina et al., 2013). The results of the current study supported the previous finding about the significantly higher level of Hsp70 in the thermotolerant *E. cyaneus*, compared with *E. verrucosus*. Nonetheless, higher critical and pejus temperatures have been estimated for this species by using physiological parameters (oxygen consumption and ventilation range) during a gradual temperature increase in a comparative study (Jakob et al., 2016). *E. cyaneus* inhabits the upper littoral zone, experiencing maximal temperature fluctuations, and reproduces in summer months at temperatures of 15−18 °C. Unlike *E. cyaneus*, the congener *E. verrucosus* is characterized by decreased thermal tolerance, and a reproduction period during the winter at ambient temperatures of 3−4 °C. The adult forms of this species mainly migrate to the deeper and colder parts of the littoral and the sublittoral zone during the warmer summer months (Weinberg & Kamaltynov, 1998). According to Bazikalova (1945), *E. verrucosus* belong to the winter-reproducing complex of Baikal amphipods, and *E. cyaneus* belong to the summer-reproducing complex. It was proposed that the summer-reproducing complex originated in Lake Baikal earlier, when the climate condition was warmer (Bazikalova, 1941; Takhteev, 2000). Recent molecular phylogenetic studies clearly demonstrate, that despite the fact that both species originated within the proto Baikal stage (30–3.5 m.y.a), *E. cyaneus* originated approximately 8 m.y.a., and *E. verrucosus* originated approximately 6 m.y.a (Mats, Shcherbakov & Efimova, 2011). The end of the proto Baikal stage was characterized by a general cooling (Mats, Shcherbakov & Efimova, 2011), which most likely led to the formation of the winter-reproducing complex. As demonstrated in this study, these two species have a strict difference in the intersexual level of Hsp70. The females of the thermosensitive *E. verrucosus* have a significantly lower Hsp70 level than the males, while the females of the thermotolerant *E. cyaneus* have similar or even slightly higher levels of Hsp70. This indicates that female *E. verrucosus* are more vulnerable to temperature increases since they breed during the winter and lack the necessary regulation of Hsp70 activation during the temperature increase. Conversely, females of the thermotolerant summer-reproducing species* E. cyaneus* activate the regulation of Hsp70 induction during their breeding to sustain summer temperatures in the upper littoral zone. This mechanism allows the females to increase the Hsp70 level during heat shock and recovery. Considering the shown species-specific differences in the molecular mechanism of Hsp70 expression (Bedulina et al., 2013), we suggest that this regulation in *E. cyaneus* can be due to higher concentrations of Hsp70 in cells, higher number of Hsp70 gene copies, and their respective protein isoforms, or more compact promoters of Hsp70 genes in this species. However, this assumption requires additional research.

To further characterize the cellular heat shock response in amphipods, proteomic analysis was carried out. This approach allows for the analysis of all of the proteins present in an organism at a given time. 2D-PAGE is a powerful tool for the investigation of proteomes. In the present study, we described, for the first time, the most abundant proteins in the proteomes of the two endemic Baikal amphipod species for both the control and heat shock groups. Because of the low similarity to public databases of the protein sequences, the sex-specific spots were not identified. According to a recent proteomic study on reproductive tissues of the freshwater amphipod species *Gammarus fossarum* Koch, 1836, the most abundant proteins in female gammaridean amphipods are the clottable proteins, vitellogenin and apolipocrustaceanin (Trapp et al., 2014). Future research is necessary to determine whether the detected spots belong to these proteins. Our results indicate the higher lability of the proteomes in the females of both of the species under heat shock and recovery. Similar results were reported for the amphipod species *Diporeia spp.* under toxic stress; a greater number of protein spots changed in the females of this species (Ralston-Hooper et al., 2011). The high number of downregulated protein spots indicate a general reaction towards heat shock, consisting of the stop of general cellular protein synthesis and the activation of stress-responses (Hochachka & Somero, 2002). Proteomic studies carried out on various marine organisms revealed the main groups of the heat shock stress-response proteome, which include protein homeostasis (molecular chaperones and proteasome subunits), anaerobic and aerobic metabolism (e.g., Krebs cycle), ROS scavenging (oxidative stress proteins), cytoskeleton (e.g., actin binding proteins) and signaling (Ras-related small GTPases) (Tomanek, 2014). Our results show that four protein spots had significantly increased optical densities in the female *E. cyaneus*. Among these, three proteins were identified as hemocyanin and one as arginine kinase. Hemocyanin has an important respiratory function in many invertebrates. In addition, some isoforms of hemocyanin act as phenoloxidase, providing an immune response function (Adachi et al., 2003). Hemocyanin of *E. verrucosus* is represented by at least 24 of the proteoforms found on the 2D gels. They were encoded by at least 7 isoforms of the transcripts from two types: an unclassified type, found only in amphipods and the *β*-type, which is typical for percarids (Crustacea: Percarida) (Bedulina et al., 2016). Abiotic stressors alter the hemocyanin isoform composition in crustaceans (DeFur, Mangum & Reese, 1990; Mangum, Greaves & Rainer, 1991; Mangum, 1997; Decker & Foll, 2000). Increased hemocyanin, under various stressors, was found in a proteomic study of the amphipod species *Diporeia spp.* and *Gammarus fossarum* (Ralston-Hooper et al., 2011; Leroy et al., 2010) and the porcelain crab *Petrolisthes cinctipes* (Randall, 1840) (Garland, Stillman & Tomanek, 2015). An elevation in hemocyanin possibly allows female *E. cyaneus* to increase the affinity of this protein for oxygen, thereby providing more energy for the protective function. However, the sensitivity of the females is still higher than that of the males, which leads to the overarching conclusion that there is an increased energy demand in the females of this species.

Arginine kinase (AK) is responsible for the reversible transfer of the high-energy phosphate from arginine phosphate to ADP to form ATP:

\begin{eqnarray*}\mathrm{Argininephosphate}+\mathrm{ADP}\Longleftrightarrow ^{\mathrm{AK}}\mathrm{Arginine}+\mathrm{ATP} \end{eqnarray*}

This enzyme is the key metabolic enzyme in crustaceans, providing a cellular respiratory function (Ellington, 2001; Strong & Ellington, 1995; Abe, Hirai & Okada, 2007). An alteration in AK was shown for many crustaceans under a variety of stressors. In the freshwater amphipod *Gammarus pulex* (Linnaeus, 1758), AK was downregulated under polychlorinated biphenyls exposure (Leroy et al., 2010). AK was upregulated in the two freshwater amphipods *Hyalella azteca* and Diporeia spp. when exposed to atrazine (Ralston-Hooper et al., 2011). A comparatively large spot of AK on the gels from the amphipods suggests that this protein is highly abundant in the proteomes of amphipods. Upregulation of AK, along with the hemocyanin subunit, indicates that there is activation of oxygen metabolism and energy acquisition in *E. cyaneus* females. Bringing together the Hsp70 and proteomic results, we can consider that *E.cyaneus* females are better equipped for an acute thermal stress response than *E.verrucosus* females, as indicated by the presence of higher energy metabolic proteins following a heat shock response together with more efficient molecular mechanisms of an Hsp70-mediated heat shock response.

## Conclusion

The results of the present study demonstrate that there is a significant difference in the intraspecific and acute thermal stress sensitivity and cellular stress-response in Baikal endemic amphipods. Further research is needed to reveal the sensitivity of other ontogenetic stages of the studied species, such as juveniles or the females of different molting stages, and the seasonal variation of thermal sensitivity. However, the current results already suggest that winter-reproducing complex of Baikal amphipods, which dominates in the littoral zone, is vulnerable to temperature increases, which are becoming more pronounced with Lake Baikal’s rapidly increasing surface temperature (Moore et al., 2009; Hampton et al., 2008; O’Reilly et al., 2015). The extent to which these environmental changes have precipitated population or community level changes is unknown. However, various works have shown a changing community structure in Lake Baikal (Katz et al., 2015; Izmest’eva et al., 2016; Timoshkin et al., 2016), where the potential for alterations of species interactions exist (Meyer et al., 2016). Because of the large variation in ecosystem responses to environmental conditions, physiological exploration and monitoring of Baikal’s stenothermic organisms, as detailed above, presents a potential mechanism for understanding fine-scale alterations long before higher level alterations are observed.

##  Supplemental Information

10.7717/peerj.2864/supp-1 Supplemental Information 1 Original Western Blot membrane stained with anti-Hsp70 and anti-actin solutions FC, Female control.

10.7717/peerj.2864/supp-2 Supplemental Information 2 Original Western Blot membrane stained with anti-Hsp70 and anti-actin solutions MC, Male control.

10.7717/peerj.2864/supp-3 Supplemental Information 3 Original Western Blot membrane stained with anti-Hsp70 and anti-actin solutions FC, Female control; FR, Female Heat Schock+Recovery.

10.7717/peerj.2864/supp-4 Supplemental Information 4 Original Western Blot membrane stained with anti-Hsp70 and anti-actin solutions FC, Female control; FR, Female Heat Schock+Recovery.

10.7717/peerj.2864/supp-5 Supplemental Information 5 Original Western Blot membrane stained with anti-Hsp70 and anti-actin solutions MC, Female control; MR, Female Heat Schock+Recovery; FC, Female control; FR, Female Heat Schock+Recovery; controlrol + Positive control (Bovine Hsp70)

10.7717/peerj.2864/supp-6 Data S1 Densitometry analysis of Western Blot Membranes and 2D-PAGE gels

## Additional Information and Declarations

### Data Availability

The following information was supplied regarding data availability: The raw data has been supplied as a Supplemental File.
