# Supplementary figures and images for: Intersexual differences of heat shock response between two amphipods (Eulimnogammarus verrucosus and Eulimnogammarus cyaneus) in Lake Baikal
Source: PeerJ. 2017 Feb 21;5:e2864. doi: 10.7717/peerj.2864 (PMC5322754; doi:10.7717/peerj.2864)

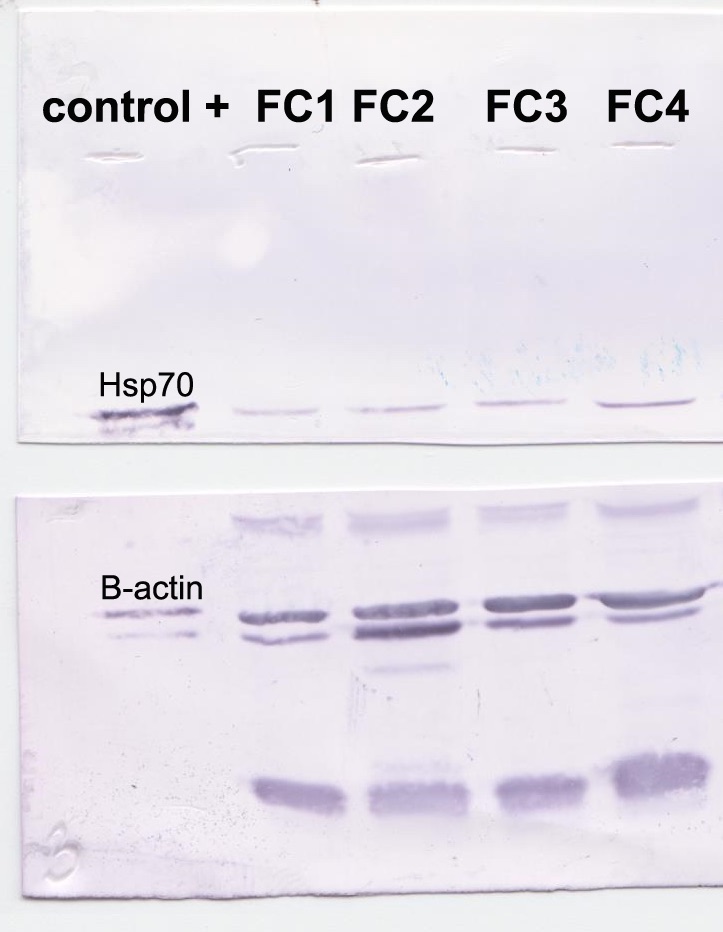

Supplement: Supplemental Information 1 — FC, Female control. [file peerj-05-2864-s001.jpg]

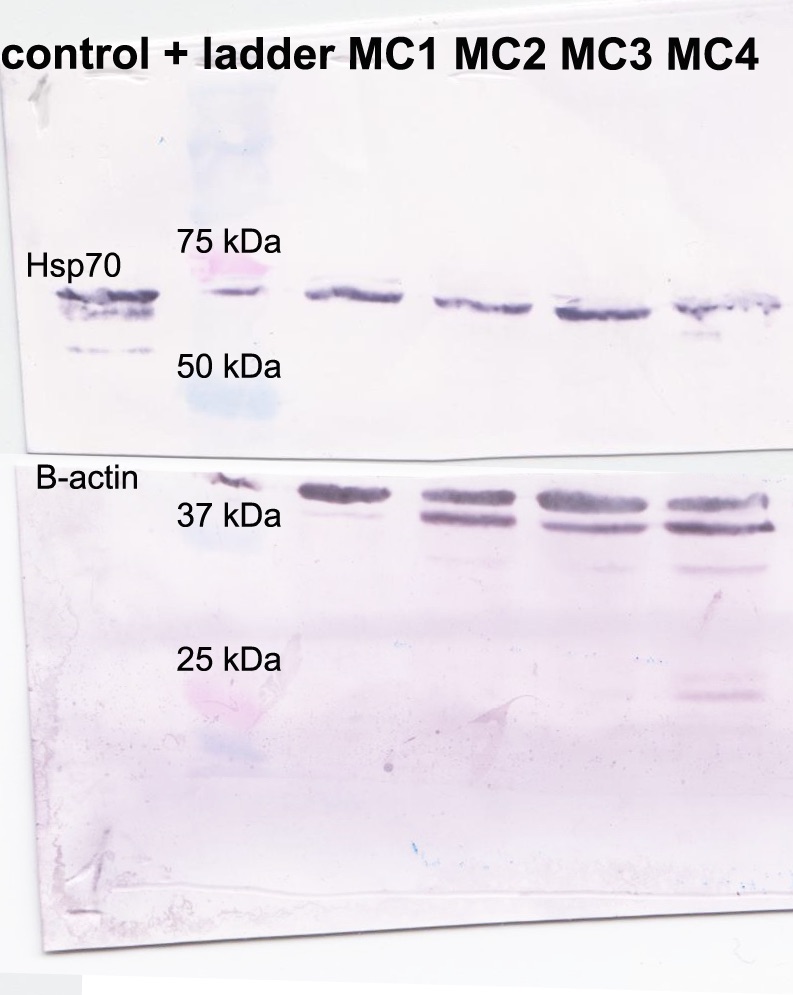

Supplement: Supplemental Information 2 — MC, Male control. [file peerj-05-2864-s002.jpg]

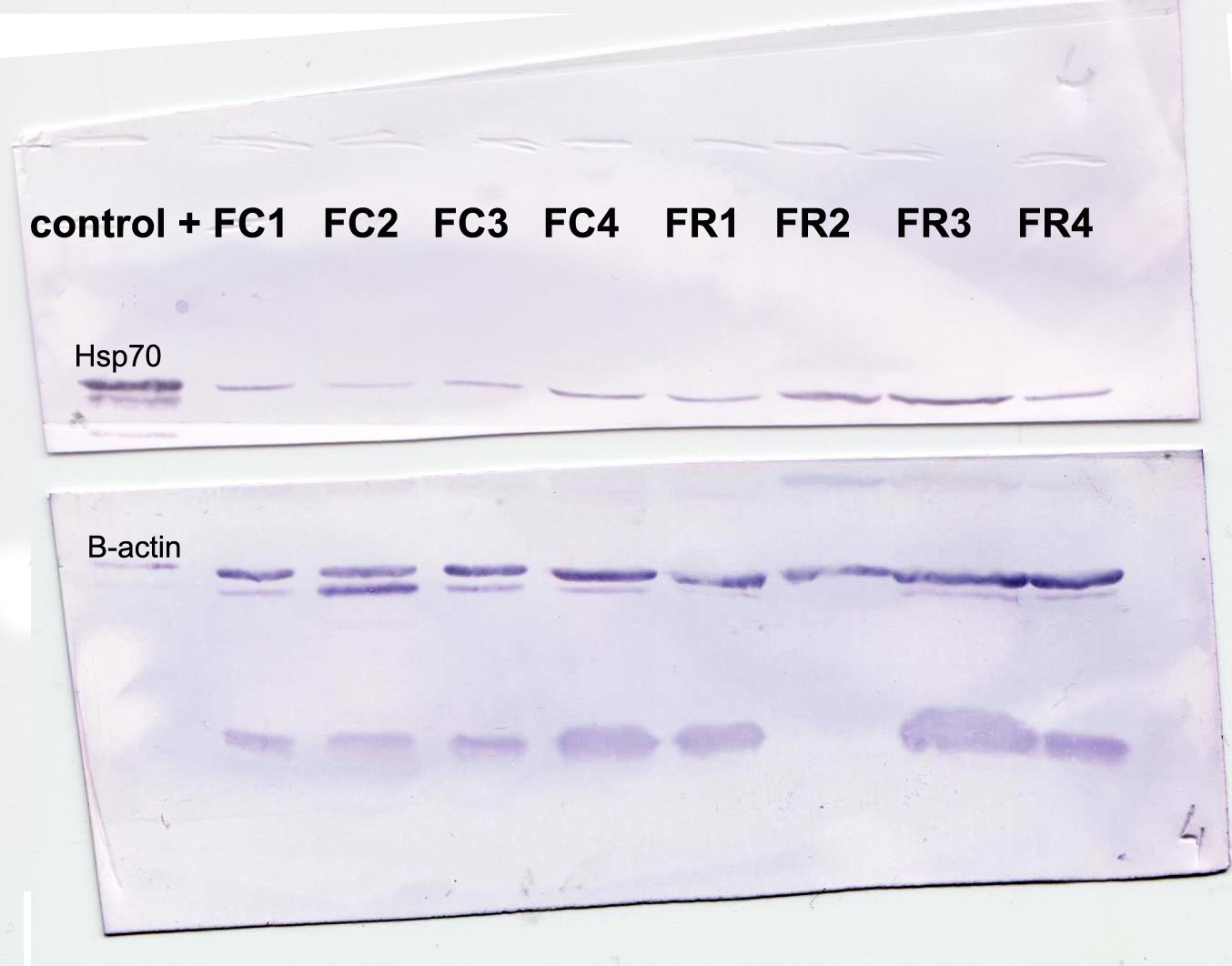

Supplement: Supplemental Information 3 — FC, Female control; FR, Female Heat Schock+Recovery. [file peerj-05-2864-s003.jpg]

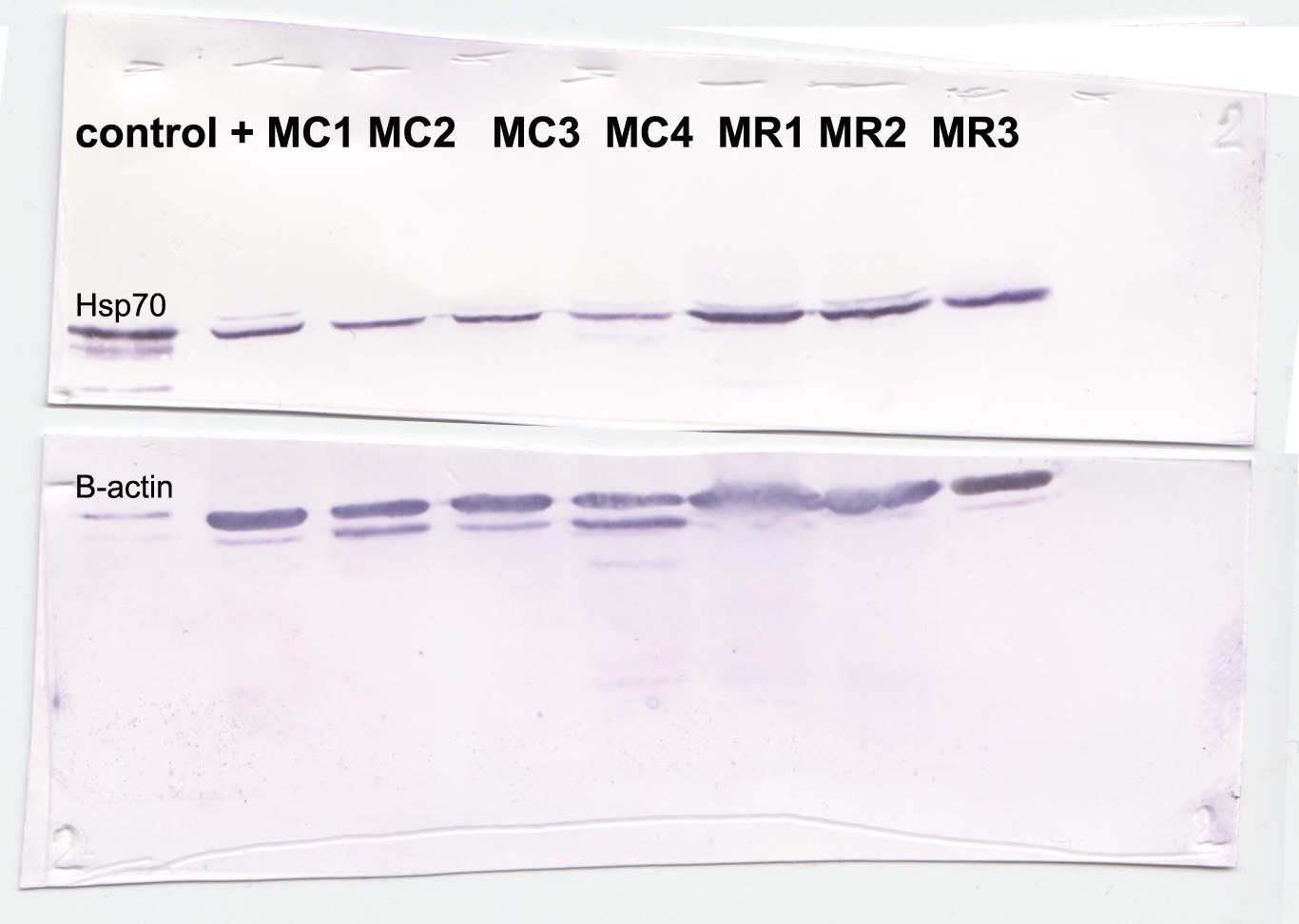

Supplement: Supplemental Information 4 — FC, Female control; FR, Female Heat Schock+Recovery. [file peerj-05-2864-s004.jpg]

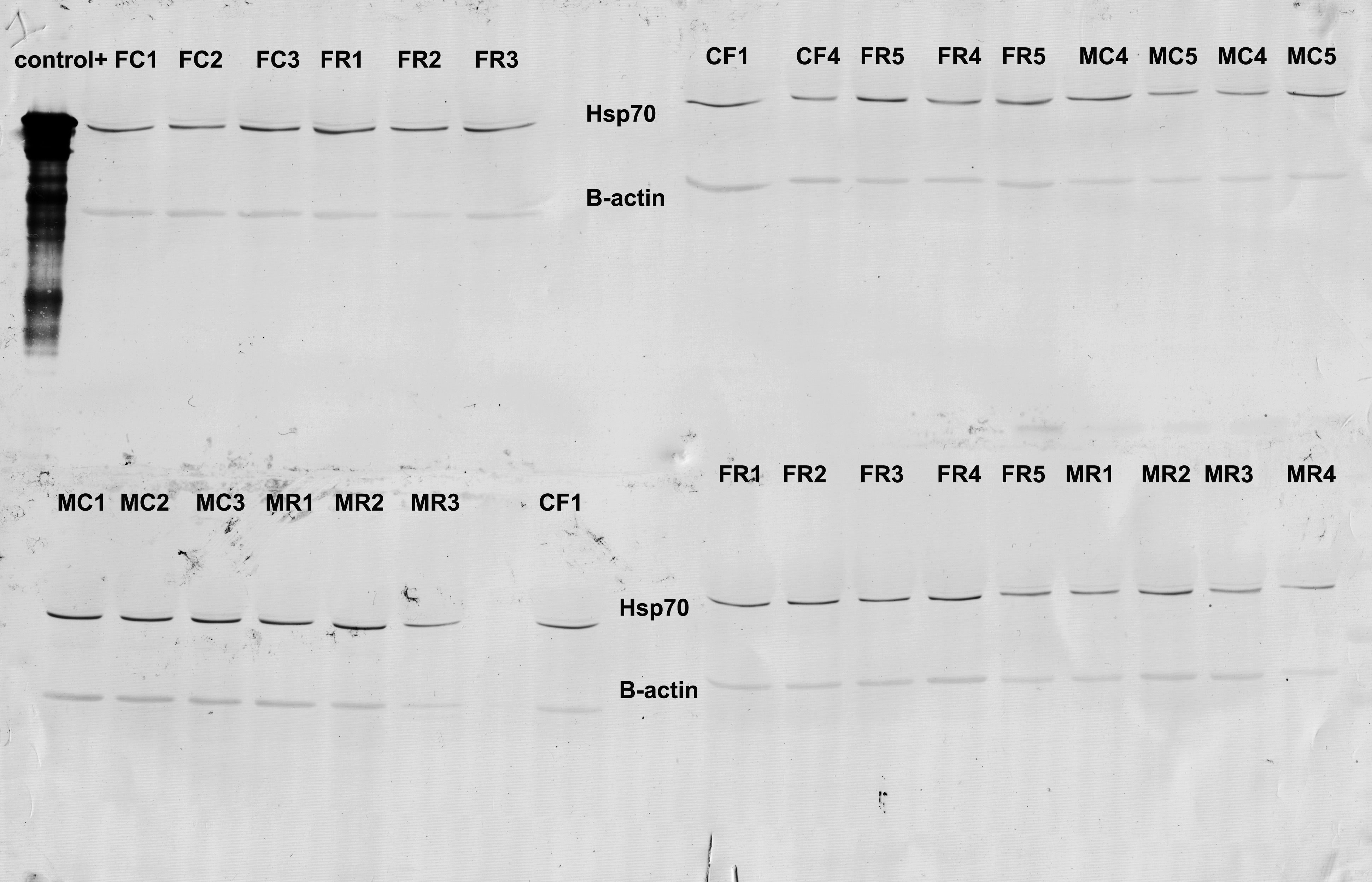

Supplement: Supplemental Information 5 — MC, Female control; MR, Female Heat Schock+Recovery; FC, Female control; FR, Female Heat Schock+Recovery; controlrol + Positive control (Bovine Hsp70) [file peerj-05-2864-s005.jpg]
